# Using the inner membrane of *Escherichia coli* as a scaffold to anchor enzymes for metabolic flux enhancement

**DOI:** 10.1002/elsc.202200034

**Published:** 2023-01-10

**Authors:** You Wang, Yushu Wang, Yuqi Wu, Yang Suo, Huaqing Guo, Yineng Yu, Ruonan Yin, Rui Xi, Jiajie Wu, Nan Hua, Yuehan Zhang, Shaobo Zhang, Zhenming Jin, Lin He, Gang Ma

**Affiliations:** ^1^ Bio‐X‐Renji Hospital Research Center Renji Hospital School of Medicine Shanghai Jiao Tong University Shanghai P.R. China; ^2^ Bio‐X Institutes Key Laboratory for the Genetics of Developmental and Neuropsychiatric Disorders (Ministry of Education) Shanghai Jiao Tong University Shanghai P.R. China; ^3^ 2012 SJTU‐BioX‐Shanghai Team for The International Genetically Engineered Machine Competition (iGEM) Shanghai Jiao Tong University Shanghai P.R. China

**Keywords:** cell membrane, fatty acid biosynthesis, metabolic flux, scaffold

## Abstract

Clustering enzymes in the same metabolic pathway is a natural strategy to enhance productivity. Synthetic protein, RNA and DNA scaffolds have been designed to artificially cluster multiple enzymes in the cell, which require complex construction processes and possess limited slots for target enzymes. We utilized the *Escherichia coli* inner cell membrane as a native scaffold to cluster four fatty acid synthases (FAS) and achieved to improve the efficiency of fatty acid synthesis in vivo. The construction strategy is as simple as fusing target enzymes to the N‐terminus or C‐terminus of the membrane anchor protein (Lgt), and the number of anchored enzymes is not restricted. This novel device not only presents a similar efficiency in clustering multiple enzymes to that of other artificial scaffolds but also promotes the product secretion, driving the entire metabolic flux forward and further increasing the gross yield compared with that in a cytoplasmic scaffold system.

## 1 INTRODUCTION

In cells, many enzymes undergo energy uptake and produce materials essential for daily life processes. A number of enzymes in a metabolic flux naturally cluster as multi‐enzyme “sequential” or “cascade” reactions, such as glycolysis and Krebs cycle [1, 2]. The existence of these natural “flow lines” indicates that clustering relevant enzymes can improve the efficiency of metabolic flux and further save biological energy.

To mimic a natural multi‐enzyme complex and to organize functional‐related enzymes, researchers developed several approaches, such as designing an artificial protein scaffold for the generation of the desired metabolic flux [3]. Using well‐characterized and widespread protein–protein interaction domains from metazoan signaling proteins (SH3‐, PDZ‐, and GBD‐binding domains), the author constructed a modular genetically encoded scaffold system, where enzyme localization was predefined and programmable. With this system, the amount of the target product increased by 77‐fold, demonstrating the advantages of artificial scaffolds [3, 4].

Nevertheless, protein and other scaffold systems with predefined artificial scaffolds are generally limited by the length of scaffolds or the number of artificially clustered modules [5, 6]. To simplify the clustering system and expand the number of enzymes that can be exerted to the system, we proposed that the inner cell membrane could be a good candidate because of its several properties. First, unlike synthesized scaffolds, the cell membrane is an innate organelle that has no amount limitation. Second, the cell membrane has a much more compact space than the cytosol, suggesting the presence of unlimited slots for scaffolding proteins. Third, the native structure of cell membrane restricts the reaction place to a two‐dimensional plane compared with discrete scaffolds, thereby facilitating the enzymatic interactions happen between several anchored proteins. Moreover, the enzymes can be organized in a 2D pattern on the membrane to further enhance the metabolic flux. The proposed membrane scaffold could be used to effectively increase the concentration of the final products near the membrane, thereby facilitating the transmembrane transportation of products and further simplifying the post‐processing procedure. Therefore, exploiting the potential of the cell membrane as a native scaffold for clustering enzyme systems is of great importance.

**PRACTICAL APPLICATION**

The membrane scaffold we developed here is an innate system without limitation on scaffold number, which will accelerate in vivo biosynthesis of high value‐added chemicals marvelously. By simple protein fusion, this kind of scaffold can load more than four enzymes to realize the synergistic catalysis of multi‐enzyme system. To produce economic biofuel which could be put into mass production, we can transform alkane‐producing membrane scaffold into autotrophic microbial cells such as cyanobacteria, where alkane could be produced and exported effectively driven only by light and carbon dioxide, which further reduces the energy expenses of heterotrophic organisms. Besides, the membrane scaffold can also be applied in other fields such as biodegradation, signal transduction, nano‐materials and so on.

**FIGURE 1:**
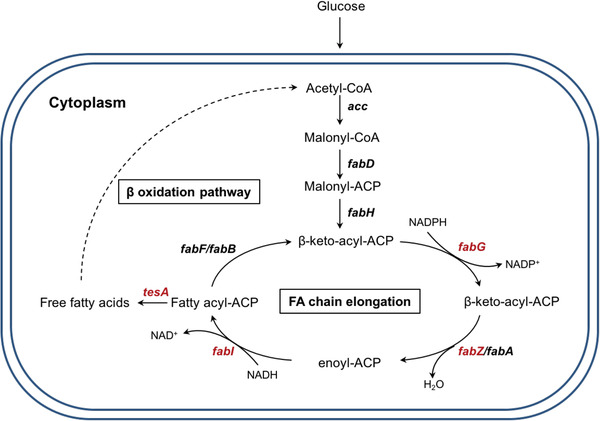
Fatty acid metabolism pathway in *E. coli*. FabA, FabB, FabD, FabF, FabG, FabH, FabI, FabZ are the main enzymes of the fatty acid biosynthesis pathway in *E. coli*. FabB, FabF, Fab G, FabI, FabZ/FabA are mainly responsible for fatty acid elongation. TesA is the enzyme that is able to release free fatty acid by hydrolysis of acyl‐ACP species. FabG, FabZ, FabI and TesA were picked as targets for manipulation in these experiments based on previous studies

To verify this concept, we selected four key enzymes of the fatty acid metabolic pathway in *Escherichia coli* and anchored them onto the inner cell membrane. *E. coli* has nine fatty acid synthases (FAS), namely FabA, FabB, FabD, FabF, FabG, FabH, FabI, FabZ, and ACP (Figure 1). Besides, TesA, a periplasmic thioesterase, can release free fatty acids (FFAs) from acyl‐ACP species. Previous studies suggested that FabG, FabI, FabZ, and TesA control the rate‐limiting steps in fatty acid biosynthesis in *E. coli* [7, 8, 9]. By conducting a systematic kinetic analysis on the fully reconstituted *E. coli* strains, Yu et al. suggested that different combinations of the molar ratios of FabZ, FabG, FabI, and TesA remarkably influenced the overproduction of fatty acids [7]. Therefore, we determined to fuse FabG, FabI, FabZ, and TesAʹ, which is a TesA mutant without a signal sequence peptide that redirects it to the cytoplasm and thus increase the accessibility of substrates to the active site [10], with phosphatidylglyceryl::prolipoprotein diacylglyceryl transferase, a well‐studied *E. coli* inner transmembrane protein [11], to confirm the feasibility of the membrane scaffold. By anchoring these enzymes, we observed increased fatty acids titer and dramatically enhanced products exportation. Collectively, our results provide novel insight into the potential application of cell membrane as a scaffold for important metabolic pathway to produce valuable bioproducts.

## 2 MATERIALS AND METHODS

### 2.1 Plasmids and strains

*E. coli* strains DH5α and BL21 (DE3) were used for cloning and protein expression, respectively. The vectors used for construction included pETDuet1, pACYCDuet1, pRSF‐Duet1, and pBAD18. All of the plasmids used in this study are shown in Table 1.

**TABLE 1:** Plasmids used in this study

Plasmids	Description	Source
pBAD18	Expression vector, Amp^R^, P_ara_, pBR322 ori	Beckwith lab
pET‐28a(+)	Expression vector, Kan^R^, P_T7_, pBR322 ori	Novagen
pETDuet1	Expression vector, Amp^R^, P_T7_, pBR322 ori	Novagen
pACYCDuet1	Expression vector, Cm^R^, P_T7_, p15A ori	Novagen
pRSFDuet1	Expression vector, Km^R^, P_T7_, RSF ori	Novagen
pET‐Ara	Modified expression vector containing two arabinose operons, Amp^R^, P_ara_, pBR322 ori	This study
pACYC‐Ara	Modified expression vector containing two arabinose operons, Cm^R^, P_ara_, p15A ori	This study
pRSF‐Ara	Modified expression vector containing two arabinose operons, Km^R^, P_ara_, RSF ori	This study
pETara‐Anchor	SsDsbA‐Bla‐Lgt‐GFP inserted into pET‐28a	This study
MA1‐1EGFP	SsDsbA‐Lgt‐GBDligand‐1EGFP inserted into BglII/XhoI sites of pET‐Ara	This study
MA2‐2EGFP	SsDsbA‐PDZdomain‐Lgt‐GBDdomain‐2EGFP inserted into EcoRI/PstI sites of pACYC‐Ara	This study
MA3‐1EGFP	SsDsbA‐PDZligand‐Lgt‐SH3ligand‐1EGFP inserted into EcoRI/PstI sites of pET‐Ara	This study
MA4‐2EGFP	SsDsbA‐Lgt‐SH3domain‐2EGFP inserted into EcoRI/PstI sites of pRSF‐Ara	This study
M‐1EGFP	SsDsbA‐Lgt‐1EGFP inserted into EcoRI/PstI sites of pET‐Ara	This study
M‐2EGFP	SsDsbA‐Lgt‐2EGFP inserted into EcoRI/PstI sites of pRSF‐Ara	This study
MA1‐FabI	SsDsbA‐Lgt‐GBDligand‐FabI inserted into BglII/XhoI sites of pET‐Ara	This study
MA2‐FabZ	SsDsbA‐PDZdomain‐Lgt‐GBDdomain‐FabZ inserted into EcoRI/PstI sites of pACYC‐Ara	This study
MA3‐FabG	SsDsbA‐PDZligand‐Lgt‐SH3ligand‐FabG inserted into EcoRI/PstI sites of pET‐Ara	This study
MA4‐TesA’	SsDsbA‐Lgt‐SH3domain‐TesA’ inserted into EcoRI/PstI sites of pRSF‐Ara	This study
M‐FabI	SsDsbA‐Lgt‐FabI inserted into BglII/XhoI sites of pET‐Ara	This study
M‐FabZ	SsDsbA‐Lgt‐FabZ inserted into EcoRI/PstI sites of pACYC‐Ara	This study
M‐FabG	SsDsbA‐Lgt‐FabG inserted into EcoRI/PstI sites of pET‐Ara	This study
M‐TesA’	SsDsbA‐Lgt‐TesA’ inserted into EcoRI/PstI sites of pRSF‐Ara	This study
CB‐FabI	GBDligand‐FabI inserted into BglII/XhoI sites of pET‐Ara	This study
CB‐FabZ	PDZdomain‐GBDdomain‐FabZ inserted into EcoRI/PstI sites of pACYC‐Ara	This study
CB‐FabG	PDZligand‐SH3ligand‐FabG inserted into EcoRI/PstI sites of pET‐Ara	This study
CB‐TesA’	SH3domain‐TesA’ inserted into EcoRI/PstI sites of pRSF‐Ara	This study
C‐FabI	FabI inserted into BglII/XhoI sites of pET‐Ara	This study
C‐FabZ	FabZ inserted into EcoRI/PstI sites of pACYC‐Ara	This study
C‐FabG	FabG inserted into EcoRI/PstI sites of pET‐Ara	This study
C‐TesA’	TesA’ inserted into EcoRI/PstI sites of pRSF‐Ara	This study

**FIGURE 2:**
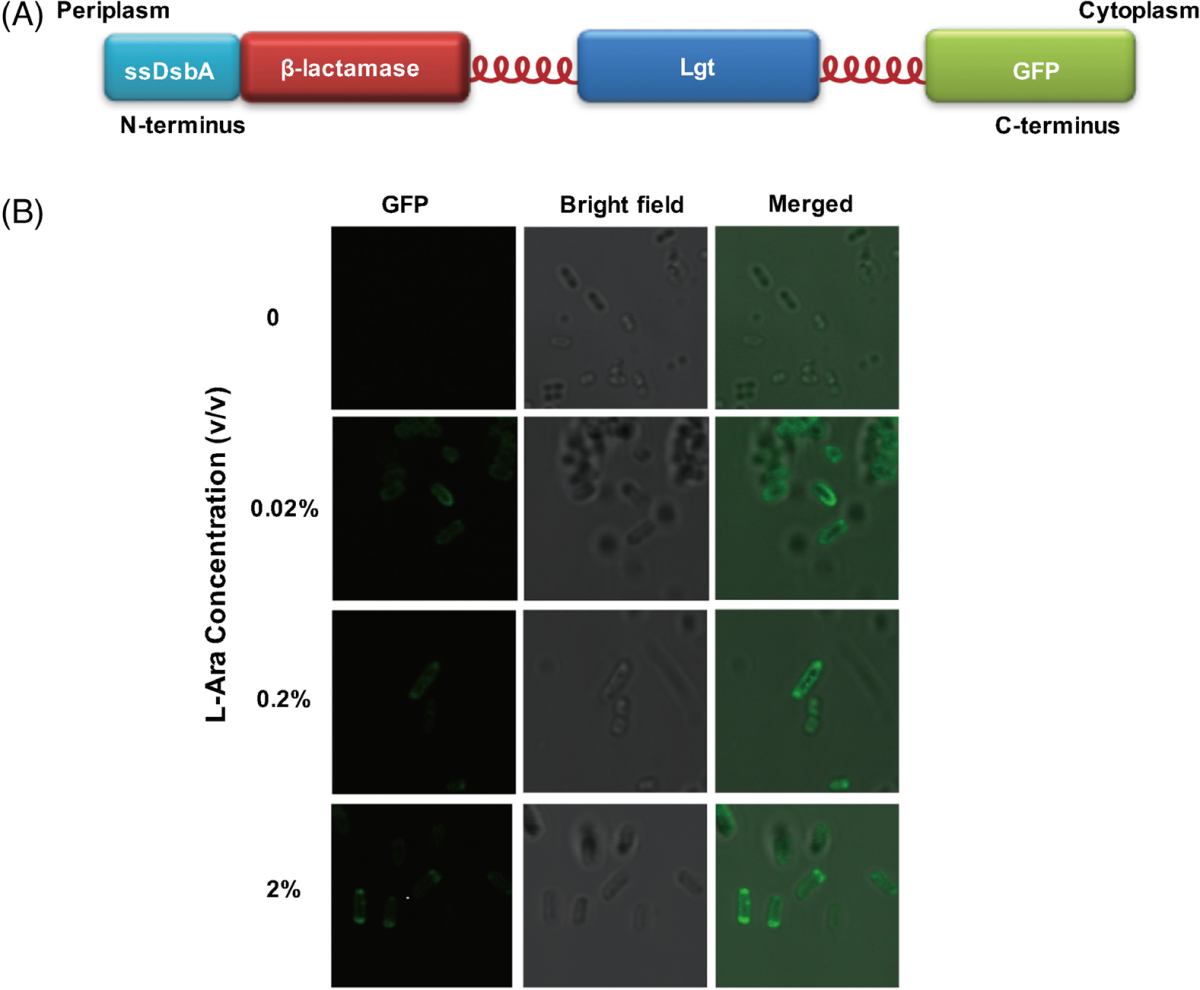
Design and verification of the membrane localization of the engineered Lgt. (A) Schematic of the engineered membrane protein. Lgt is used as a scaffold to carry functional groups (β‐lactamase and EGFP as examples) to the membrane. (B) Membrane localization is verified by confocal microscopy

**FIGURE 3:**
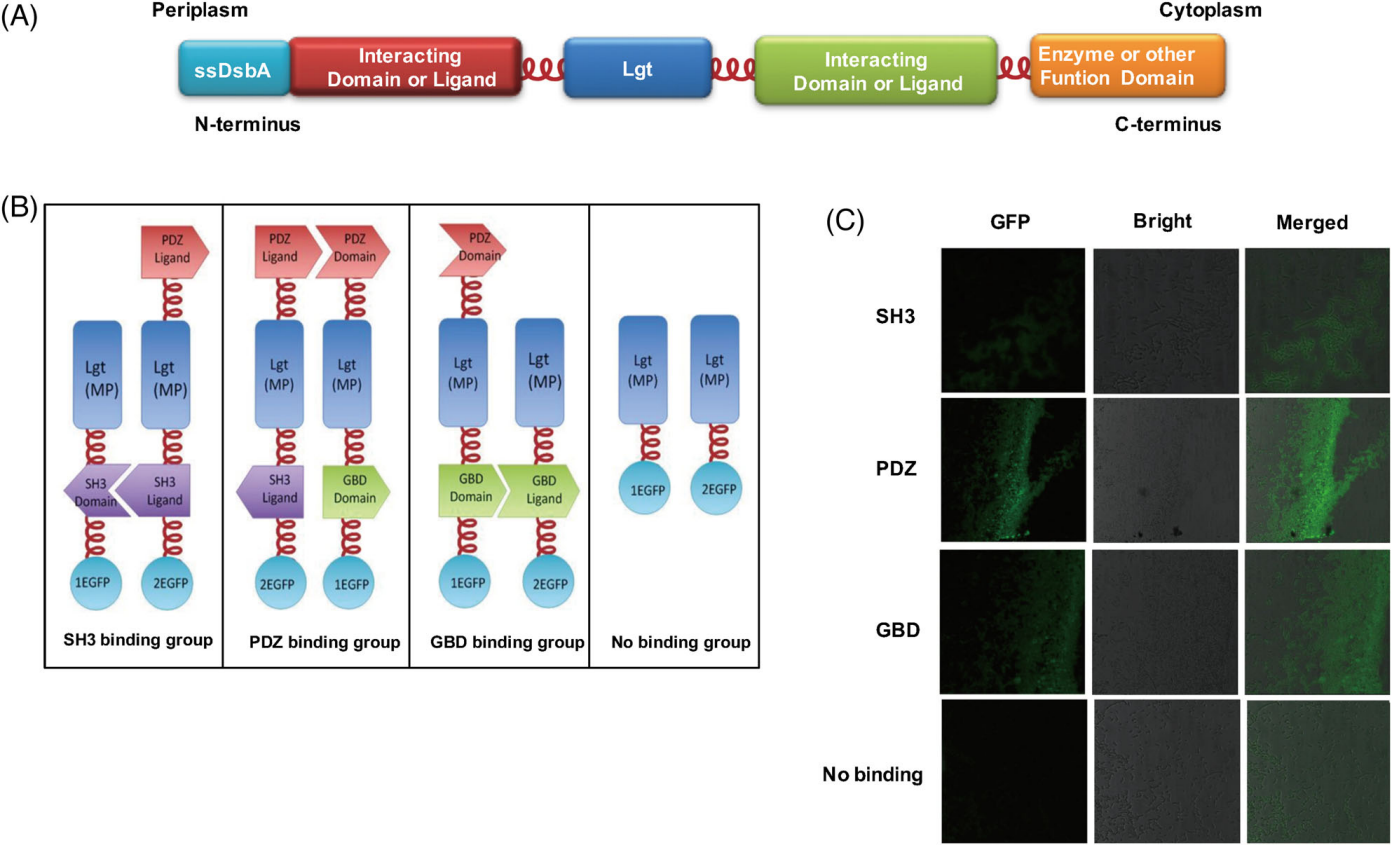
Design and verification of the artificial membrane clustering. (A) Schematic of the engineered membrane protein. Protein interaction domains are fused with the ends of Lgt. (B) Schematic of the four groups of engineered membrane protein. The latter three groups use SH3, PDZ, and GBD interactions, respectively. The No binding group has no interaction domains. Split EGFP is fused with the N‐terminus of the membrane protein to verify the protein interactions. (C) The protein interactions in different groups are verified by confocal microscopy. The detected EGFP fluorescence indicates the interactions between designed proteins

The arabinose operon from pBAD18 was amplified and cloned twice into pETDuet1, pACYCDuet1, and pRSF‐Duet1 to replace the original T7 operon, thereby producing pET‐Ara, pACYC‐Ara, and pRSF‐Ara. Each vector contained two copies of arabinose operons. For the verification of membrane localization, the DNA fragment containing the N‐terminal DsbA signal sequence, followed by the genes of β‐lactamase, phosphatidylglyceryl::prolipoprotein diacylglyceryl transferase (Lgt), and GFP was cloned into pET‐Ara (Figure 2A). For the verification of artificial clustering, the DNA fragment comprising a DsbA signal sequence (ssDsbA), one type of interacting protein, Lgt, and a split EGFP was cloned into arabinose operon (Figures 3A and 3B). A flexible linker FL3 (ACTAGAGCTGAGGCCGCCGCAAAAGAAGCAGCAGCTAAGG AAGCTGCGGCGAAG) was introduced between crucial protein parts to ensure their proper functioning.

Four groups of engineered fatty acid‐related enzymes were used to verify our design (Figure 3A). The genes involved in the membrane binding FAS (MBF), membrane FAS (MF), and cytoplasmic binding FAS (CBF) groups were cloned into pET‐Ara, pACYC‐Ara, and pRSF‐Ara, respectively. The genes in the cytoplasmic FAS (CF) group were cloned into pET‐Ara. The MBF group contained cytoplasmic and periplasmic protein interaction domains to cluster engineered proteins. The enzymes in the MF group were directly fused with the C‐terminus of Lgt. The enzymes in the CBF group were directly fused with the protein interaction domains and expressed in the cytoplasm. The CF group comprised the enzymes expressed in the cytoplasm. In each group, the FabI and FabZ proteins or the fusion proteins were cloned into two arabinose operons in pET‐Ara. The FabG and TesAʹ proteins or the fusion proteins were cloned into two arabinose operons in pRSF‐Ara.

### 2.2 Cell culture conditions for fatty acid biosynthesis

Cells carrying different constructs were incubated in 5 ml of LB medium supplemented with antibiotics and cultured overnight at 37°C. Overnight cell culture (3% [v/v]) was added to a 250 ml flask containing 50 ml of LB medium supplemented with 15 g•L^−1^ glucose and then cultivated at 37°C at a frequency of 150 rpm. Antibiotics were added to maintain the plasmids in the recombinant strains: ampicillin (100 mg/L), kanamycin (50 mg/L), or chloramphenicol (12.5 mg/L). The cultures were induced by adding 0.2% (w/v) L‐arabinose at OD600 = 0.6 and the samples were collected at 20 h post‐induction for fatty acid analysis.

### 2.3 Free fatty acid extraction and measurement

Cell culture samples (20 ml; three replicates for each sample) were centrifuged at 8000 rpm for 10 min to separate the cell‐associated fatty acids from the extracellular fatty acids. Fatty acid extraction was performed as previously described [12]. The fatty acids extracted from the supernatant were analyzed through gas chromatography–mass spectrometry (GC‐7890B, MS‐5975C, Agilent, USA) which was equipped with an HP‐5 MS column (30 m × 0.32 mm; film thickness of 0.25 mm). Helium was used as a carrier gas. The temperatures of the injector and the detector were 250 and 280 °C, respectively. The GC elution conditions were as follows: 100 °C as the starting temperature for 5 min, 15 min ramp to 250 °C, and 250 °C held constant for 5 min. All of the samples were spiked with pentadecanoic acid (C15) as an internal standard. The growth of the cells and the analysis of the fatty acid products were repeated thrice.

## 3 RESULTS

### 3.1 Localizing target enzymes to *E. coli* inner membrane

The membrane scaffold system has two indispensable elements. The Phosphatidylglyceryl::Prolipoprotein Diacylglyceryl transferase (Lgt), an *E. coli* transmembrane protein, is selected as the anchor module. Target enzymes can be fused with its N‐terminus as a periplasmic enzyme or C‐terminus as a cytoplasmic enzyme. Aside from Lgt, the signal recognition particle (SRP)‐dependent signaling sequence of DsbA (abbreviated as ssDsbA), is necessary to orient the combined proteins onto the inner membrane of *E. coli* [13]. To verify the function of the membrane anchor part, we fused β‐lactamase and EGFP with the N‐terminus of Lgt and the C‐terminus, respectively (Figure 2A) [14]. The expression plasmid containing this construct was transformed to BL21 (DE3) and induced by L‐arabinose (Figure 2B). The BL21/pETara‐Anchor strain exhibited a clear green fluorescence on the cell margin under the laser confocal microscope, confirming that the membrane anchor part was correctly localized. To further characterize the membrane anchor and define the optimal induction condition, we compared the growth phenotype of the BL21/pETara‐Anchor strain at different inducer and antibiotic levels (Figure S1) and quantitatively tested its growth status under different concentration combinations of L‐arabinose (ranging from 0 to 0.2% w/v) and ampicillin (ranging from 0 to 200 μg/ml) (Figure S2). The results revealed that 0.2% w/v L‐arabinose induction was the optimal condition for membrane protein expression. In generally speaking, by fusing with the membrane anchor, the target proteins performed normal functions regardless of their periplasmic or cytoplasmic locations.

### 3.2 Clustering target enzymes on the membrane through mammalian protein–protein interaction domains and ligands

Given that the cell membrane has a more compact space than the volume of the cytoplasm, we chose the inner cell membrane of *E. coli* as the scaffold. However, whether the proteins anchored onto the membrane can be clustered to accelerate the metabolic flux, as in the case of other artificial scaffolds, remains uncertain. To confirm that the membrane scaffold equipped with metazoan interacting proteins could be clustered, we conducted fluorescence complementation assay (Figures 3A and 3B) [3, 15]. First, protein–protein interaction domains and ligands from metazoan cells (mouse SH3 and PDZ domains and rat GBD domain) were utilized on the basis of the combination of the protein domains and their corresponding cognate ligands to rationally assemble and arrange enzymes onto the inner membrane of *E. coli*. These three groups of interacting proteins were fused with the N‐ or C‐terminus of Lgt to create the desired protein complexes on the membrane (Figure 3B).

In the fluorescence complementation assay, the fluorescent protein EGFP was split into two halves [16] (namely 1EGFP and 2EGFP), and the proteins that were postulated to cluster were fused with the unfolded complementary fragments of EGFP and expressed in *E. coli*. The interaction between protein domain and the ligand brought the fluorescent fragments within proximity, allowing the reporter protein to restore its native 3D structure and emit a fluorescent signal. Therefore, fluorescence could be observed if an interaction occurred between 1EGFP and 2EGFP. Otherwise, no fluorescence could be detected. The split EGFP parts were fused with Lgt without interacting proteins as the negative control or fused with Lgt interaction groups to test the protein clustering (Figure 3B). In Figure 3C, the detected green fluorescence signal implied that all of the three groups of Lgt fusion proteins successfully developed a functional EGFP on the membrane, demonstrating that the membrane proteins with interacting proteins could dimerize with one another. Thus, we could easily use the inner membrane as a scaffold to cluster target proteins by recruiting Lgt, which is the native membrane protein of *E. coli*, mammalian interacting proteins (SH3, PDZ, and GBD), and target enzymes.

### 3.3 Clustering fatty acid synthesis enzymes on the membrane facilitates bio‐product exporting

**FIGURE 4:**
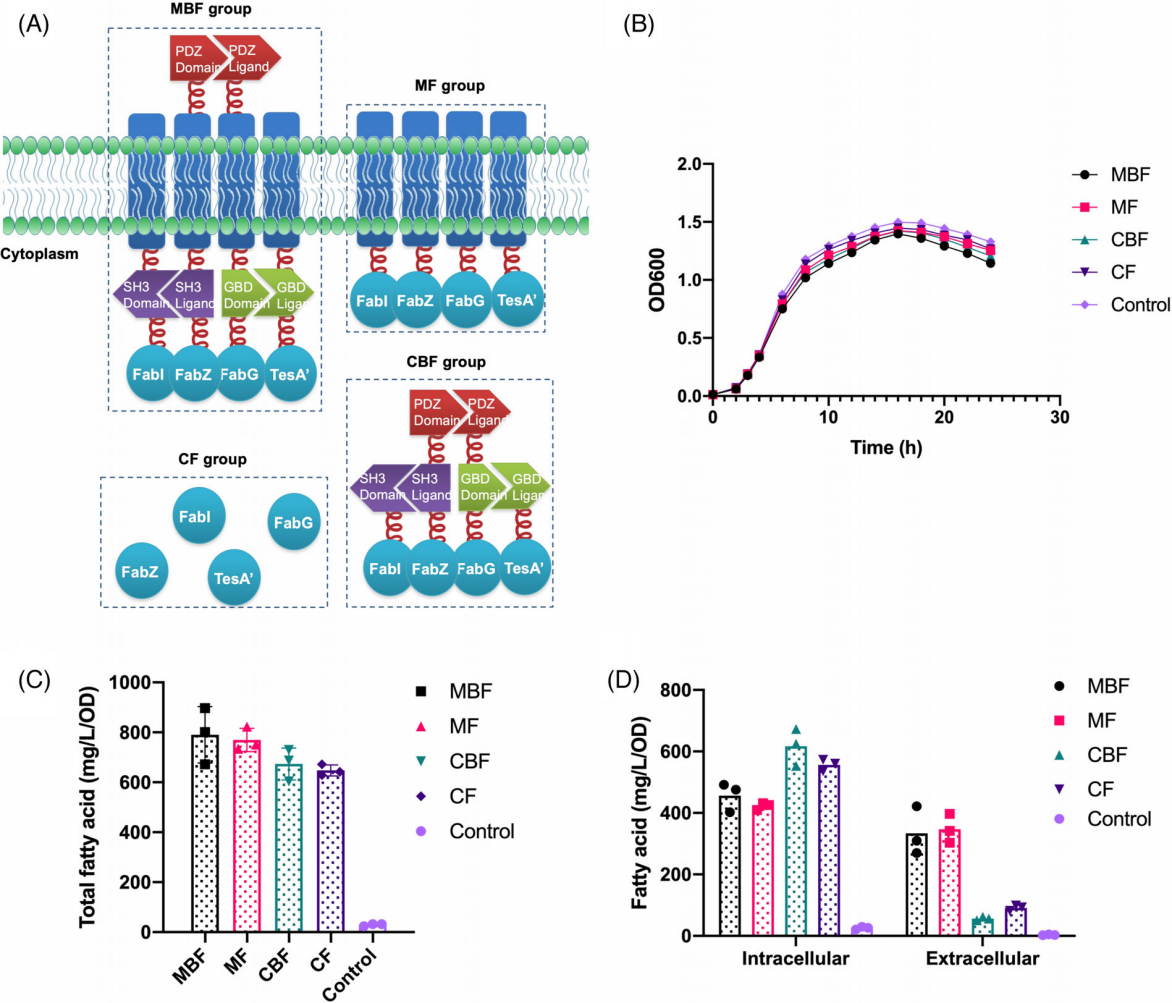
Clustering fatty acid metabolism enzymes on the membrane enhances the product yield and secretion. (A) Schematic of the four strategies of clustering enzymes. The MBF Group uses protein interaction domains to cluster target enzymes on the membrane. The MF Group directly localizes target enzymes on the membrane. The CBF Group utilizes protein interaction domains to cluster target enzymes in the cytoplasm. The CF Group refers to the strains overexpressing target enzymes freely in cytosol. (B) Growth pattern of fatty acid‐producing strains. (C) Total fatty acid extracted from the cell expressing each groups of engineered enzymes. (D) The intracellular and extracellular fatty acid titer produced by different groups. BL21 (DE3) strain were taken as the control group. Data shown in (B‐D) are the mean ± SD from three biological triplicates

Considering the successful construction of the membrane scaffold, we next introduced FabI, FabZ, FabG, TesAʹ, the four crucial FAS, in our membrane scaffold system to demonstrate the applicability and efficiency. Four groups of fatty‐acid‐overproducing strains with different scaffolding patterns were developed. The MBF group had four cascaded FAS enzymes on the inner membrane with the help of interacting proteins, whereas MF group only comprised four FAS enzymes anchored on the membrane without the aid of interacting proteins. The enzymes in the CBF group were directly fused with the protein interaction domains and expressed in the cytoplasm, while the CF group comprised the enzymes expressed freely in the cytoplasm (Figure 4A). To investigate whether the membrane‐anchored enzymes would influence the growth pattern of cell chasis, we first monitored the growth curve by measuring OD value at 600 nm, the blank BL21 (DE3) strain was taken as control group. We found that all the fatty‐acid‐producing strains grew slower than WT, amongst them, the MBF group presented the slowest growth rate, implying the potential stress caused by enzymes anchoring on cell membrane.

The cell density of engineered strains was inclined to descend after 18 h‐cultivation, so we decided to set the cultivation time for 20 h, then the intra‐ and extracellular fatty acids were extracted and measured by GC‐MS. The total fatty acid content (refers to the pool of intracellular plus extracellular pools) produced by anchoring the enzymes onto the membrane in the MBF (790.15 mg/L/OD) and MF (769.43 mg/L/OD) groups was more than that obtained by simply overexpressing the enzymes in the cytoplasm (673.43 mg/L/OD in CBF and 647.69 mg/L/OD in CF), while the WT group only produced 29.17 mg/L/OD total FAs (Figure 4C). However, the total fatty acids obtained by simply anchoring the enzymes on the membrane in MF group was similar to that produced by clustering enzymes in MBF group, which indicated that the inner membrane, in contrast to the cytoplasm, likely retained the membrane‐anchored proteins in a relatively restrained zone, resulting in effects similar to those of clustering proteins. Thus, we performed fluorescence complementation or fluorescence resonance energy transfer experiments to verify whether simply anchoring enzymes on the membrane can cluster proteins. However, no positive results were observed (Figure 3C), which is possibly due to the enzymes anchored on the membrane were sufficiently close with one another to generate cascading effects and enhance the metabolic flux, but not close enough to be detected through fluorescence complementation or fluorescence resonance energy transfer experiments.

In addition, by analyzing the fatty acid distribution, we found that anchoring the enzymes on the membrane remarkably changed the ratio between the products in the cell and in the medium. The amount of the fatty acid products exported by the membrane groups (MBF group produced 333.57 mg/L/OD extracellular fatty acids while MF group produced 347.25 mg/L/OD) was significantly higher than that obtained by the cytoplasm groups did (56.44 and 91.16 mg/L/OD in CBF and CF groups, respectively) (Figure 4D). For both membrane‐anchor groups, 42% and 45% of total FFAs were secreted to the culture media respectively, while only about 10% FFAs secreted in cytoplasmic expression system. Together, our results demonstrated that simply anchoring the target enzymes on the membrane without introducing interacting proteins can enhance the metabolic flux as described in other artificial scaffold systems [5, 6], and the membrane scaffold system could serve to facilitate the metabolic reaction in term of the priority to product exportation. As high level of fatty acids in the culture media makes it easier to obtain and purify product, our membrane scaffold device showed huge potential for industrialized production.

## 4 DISCUSSION

Organisms naturally cluster related enzymes to improve the efficiency of the whole metabolism pathway and save energy. On the basis of this concept, researchers developed different systems to cluster target enzymes. Previous studies mainly focus on developing protein, RNA, or DNA scaffolds as constitutive assemblies carrying enzymes and have successfully increased product yields. Dueber et al. constructed an artificial compartment in *E. coli* by protein‐protein interaction domains to introduce mevalonate biosynthetic pathway, achieved 77‐fold improvement in product titer [4]. Müller et al. separately fixed Glucose Oxidase (GOX) and Horseradish Peroxidase (HRP) with DNA scaffolds and successfully improved the response efficiency [17]. Delebecque et al. applied the RNA scaffold compartment system to hydrogen production, which greatly increased hydrogen yield [18]. Although these synthetic scaffolds are useful platforms for multi‐enzyme assembly, full‐length and functional protein scaffolds may be difficult to express as the complexity of multienzyme systems increases. Besides, the unstable RNA structure hinders the application of RNA scaffolds, while DNA fragments will be cleaved by native nucleases and lose their original structure. Therefore, the robust and dynamic control of the metabolic flux remains a big challenge.

Phosphatidylglycerol::Prolipoprotein Diacylglyceryl Transferase (Lgt) is an inner membrane enzyme that catalyzes the first reaction of the three‐step post‐translational lipid modification in both Gram‐negative and Gram‐positive bacteria [19]. With a periplasmic head (N‐terminus) and a cytoplasmic tail (C‐terminus) and seven transmembrane segments, Lgt could be a perfect module to anchor interested enzymes onto the inner membrane. Theoretically, the anchored enzymes will be oriented to the cell membrane after being translated, and the enzyme distribution is restricted to the 2D membrane plane rather than randomly diffused throughout the cytoplasm, which favors the multi‐enzyme‐mediated cascade reaction. By simply fusing two functional proteins (namely β‐lactamase and GFP) on both ends of Lgt (Figure 2), we demonstrated that Lgt could be engineered as the membrane anchor part to fix enzymes in periplasm and cytoplasm space of *E. coli*.

Naturally, as there is no compartment in prokaryotic cells, enzymes involved in a biochemical pathway diffuse all over the cytoplasm. Intermediates produced from one enzyme are unable to be passed efficiently to the next one due to spatial obstacles. If such enzymes could be attached to engineered membrane proteins which constitutively assembled together, the catalytic reactions were likely to proceed more smoothly. To rationally assemble and arrange enzymes onto inner membrane of *E.coli*, protein‐ligand interaction system in metazoan cells (SH3 domain [20], PDZ domain [21], GBD domain [22]) was applied in our membrane anchor to assemble enzymes because theses peptide motifs were inclined to assemble with their cognate adaptor domains. In fluorescence complementation assay, protein domain and its cognate ligand were separately fused to two unfolded complementary fragments of EGFP and expressed in *E.coli*. Results proved that these interaction systems had brought the split EGFP within proximity, allowing the reporter protein to restore its native three‐dimensional structure and emit green fluorescent signal (Figure 3C). Therefore, it is inferred that a series of enzymes involved in sequential reactions could be swiftly and orderly organized on the membrane by our membrane scaffold.

As an important platform compound, FFAs are widely used in energy and chemical industries. Their high energy density and low water solubility make them promising alternatives to fossil fuel as transportation fuels. Initial fatty acid biosynthesis in *E.coli* is catalyzed by a series of nine enzymes and the final release of FFAs is catalyzed by a thioesterase via hydrolysis of acyl‐ACP species [23]. Here, we recruited TesA’ (truncated TesA) and three reductive enzymes (FabG, FabZ and FabI) in membrane scaffold test because previous studies showed moderate overexpression of these enzymes gave rise to elevated fatty acid productivity and turnover rate in *E. coli* [24]. As the efficiency of fatty acid synthesis was distinctly improved by artificially engineering membrane scaffolding patterns (Figure 4C), the feasibility of membrane scaffold to cluster multiple enzymes and further enhance metabolic flux was confirmed. Nevertheless, the clustered membrane‐anchored group (MBF) produced similar level of total fatty acid with the freely membrane‐anchored group (MF), which did not meet our expectations. As T7, the strong promoter, was used to control the expression of all the fatty acid biosynthetic genes in our constructs, the amount of these four enzymes distributed on inner membrane could be enough to form “metabolite microdomains” in the absence of interaction proteins. In such area, most intermediates are rapidly converted to products before escaping from the multienzyme assembly because the concentration of metabolites increases locally and is stable and persistent at steady state [25]. That could be the reason of similar fatty acid productivities shown in MBF and MF sets. According to that, although the number of clustered enzymes in membrane scaffold was limited owing to the available interacting proteins, the recruitment of interacting proteins might be indispensable for certain metabolic pathways, which would further reduce the complexity of device construction.

The random diffusion of small molecules through the cell membrane was slow and consistent with the concentration difference across the membrane. FFAs near the membrane would generate a potential concentration gradient that favoring passive diffusion of FFAs out of the cells [26]. Moreover, removal of fatty acyl‐ACP from the reaction can shift the chemical equilibrium, according to the Le Châtlier principle, to accelerate the accumulation of fatty acids. Our results showed that the products accumulated near the cell membrane when enzymes were anchored on the membrane, resulting in an increased local concentration. Such increase triggered the product molecules to diffuse outside through the cell membrane, thereby remarkably increasing the product titer in the medium (Figure 4D). The yield produced by the system with anchored enzymes on the membrane was higher than that obtained by the clustered enzymes in the cytoplasm presumably because of the continuous secretion of products. It is worth noting that more than half of fatty acid produced were retained in cell, indicating the deficient hydrolysis of fatty acyl‐ACPs, which means enhancing the efficiency of thioesterase could further promote fatty acid biosynthesis and exportation. In general, our membrane scaffold device facilitated the exportation of products, promoted the metabolic flux, and simplified the post‐processing work of the desired products.

**FIGURE 5:**
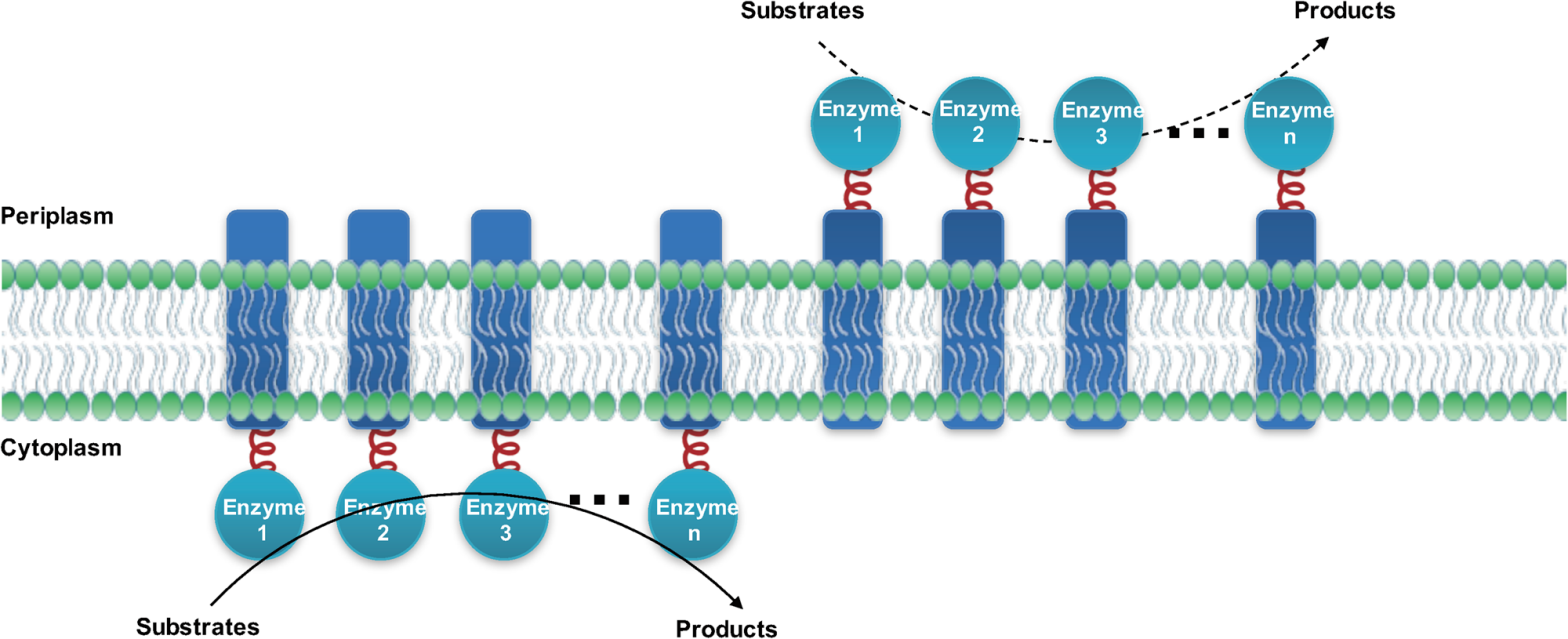
Summary of applications of membrane scaffold system. Potentially, unlimited number of target enzymes can be fused to Lgt and clustered on the membrane. The enzymes can be either presented in the cytoplasm site or the periplasm site. In the cytoplasm site, enzymes can utilize substrates produced in the cell, and the products could pass the cell membrane by diffusion. In the periplasm site, enzymes can utilize the substrates added in the culture and directly release products into the culture

In conclusion, the novel membrane scaffold system has been demonstrated to effectively enhance the metabolic flux by anchoring a series of enzymes involved in fatty acid synthesis pathway onto the native inner membrane. The construction process is simplified as fusing target enzymes to the N‐terminal or the C‐terminal of the membrane anchor protein (Lgt), and the number of the fused enzymes are not limited in theory (Figure 5). Potentially, proper enzymes can be anchored in the periplasm and utilize substrates from medium to make target products. For instance, fusing Aldehyde Decarbonylase (ADC) with N‐terminus of Lgt will probably favor the decarboxylation of long chain fatty aldehyde produced in cytosol to yield more alkanes. Moreover, the membrane scaffold not only shows similar enzymes clustering effects as other artificial scaffolds, but also boosts the products exportation, driving the metabolic flux to the positive direction and resulting in further increased final yield compared to the cytoplasm scaffolding system. In the near future, we propose to optimize the proportion of different enzymes assembled on cell membrane by synthetic biology tools, which will improve the synergistic catalysis of multiple enzymes and eventually enhance the productivity of interested products.

## CONFLICT OF INTEREST

The authors have declared no conflicts of interest.

## Supporting information

Supporting Information

## Data Availability

The data that support the findings of this study are available from the corresponding author upon reasonable request.

## References

[1] Plaxton WC . The organization and regulation of plant glycolysis. Annu Rev Plant Physio Plant Mol Bio. 1996;47(1):185‐214. 10.1146/annurev.arplant.47.1.185 15012287

[2] Thauer RK . Citric‐acid cycle, 50 years on. Modifications and an alternative pathway in anaerobic bacteria. Eur J Biochem. 1988;176(3):497‐508. 3049083 10.1111/j.1432-1033.1988.tb14307.x

[3] DeLisa MP , Conrado RJ . Synthetic metabolic pipelines. Nat Biotechnol. 2009;27(8):728‐729. 19668178 10.1038/nbt0809-728

[4] Dueber JE , Wu GC , Malmirchegini GR , et al. Synthetic protein scaffolds provide modular control over metabolic flux. Nat Biotechol. 2009;27(8):753‐U107. 10.1038/nbt.1557 19648908

[5] Hirakawa H , Haga T , Nagamune T . Artificial protein complexes for biocatalysis. Top Catal. 2012;55(16‐18):1124‐1137.

[6] Proschel M , Detsch R , Boccaccini AR , Sonnewald U . Engineering of metabolic pathways by artificial enzyme channels. Front Bioeng Biotech. 2015;3:168. 10.3389/fbioe.2015.00168 PMC4617052 26557643

[7] Yu X , Liu T , Zhu F , Khosla C . In vitro reconstitution and steady‐state analysis of the fatty acid synthase from *Escherichia coli* . Proc Natl Acad Sci USA. 2011;108(46):18643‐18648. 22042840 10.1073/pnas.1110852108 PMC3219124

[8] Heath RJ , Rock CO . Enoyl‐acyl carrier protein reductase (fabI) plays a determinant role in completing cycles of fatty acid elongation in *Escherichia coli* . J Biol Chem. 1995;270(44):26538‐26542. 7592873 10.1074/jbc.270.44.26538

[9] Heath RJ , Rock CO . Roles of the FabA and FabZ beta‐hydroxyacyl‐acyl carrier protein dehydratases in *Escherichia coli* fatty acid biosynthesis. J Biol Chem. 1996;271(44):27795‐27801. 8910376 10.1074/jbc.271.44.27795

[10] Ferreira R , Teixeira PG , Siewers V , Nielsen J . Redirection of lipid flux toward phospholipids in yeast increases fatty acid turnover and secretion. Proc Natl Acad Sci USA. 2018;115(6):1262‐1267. 29358378 10.1073/pnas.1715282115 PMC5819412

[11] Pailler J , Aucher W , Pires M , Buddelmeijer N . Phosphatidylglycerol::prolipoprotein diacylglyceryl transferase (Lgt) of *Escherichia coli* has seven transmembrane segments, and its essential residues are embedded in the membrane. J Bacteriol. 2012;194(9):2142‐2151. 22287519 10.1128/JB.06641-11 PMC3347048

[12] Varenne S , Lazdunski C . Effect of distribution of unfavourable codons on the maximum rate of gene expression by an heterologous organism. J Theor Biol. 1986;120(1):99‐110. 3528671 10.1016/s0022-5193(86)80020-0

[13] Schierle CF , Berkmen M , Huber D , et al. The DsbA signal sequence directs efficient, cotranslational export of passenger proteins to the *Escherichia coli* periplasm via the signal recognition particle pathway. J Bacteriol. 2003;185(19):5706‐5713. 13129941 10.1128/JB.185.19.5706-5713.2003 PMC193964

[14] Skretas G , Georgiou G . Simple genetic selection protocol for isolation of overexpressed genes that enhance accumulation of membrane‐integrated human G protein‐coupled receptors in *Escherichia coli* . Appl Environ Microb. 2010;76(17):5852‐5859. 10.1128/AEM.00963-10 PMC2935035 20639362

[15] Hu CD , Kerppola TK . Simultaneous visualization of multiple protein interactions in living cells using multicolor fluorescence complementation analysis. Nat Biotechnol. 2003;21(5):539‐545. 12692560 10.1038/nbt816 PMC1820765

[16] Barnard E , Timson DJ . Split‐EGFP screens for the detection and localisation of protein‐protein interactions in living yeast cells. Methods Mol Biol. 2010;638:303‐317. 20238279 10.1007/978-1-60761-611-5_23

[17] Muller J , Niemeyer CM . DNA‐directed assembly of artificial multienzyme complexes. Biochem Biophys Res Commun. 2008;377(1):62‐67. 18823945 10.1016/j.bbrc.2008.09.078

[18] Delebecque CJ , Lindner AB , Silver PA , Aldaye FA . Organization of intracellular reactions with rationally designed RNA assemblies. Science. 2011;333(6041):470‐474. 21700839 10.1126/science.1206938

[19] Mao G , Zhao Y , Kang X , et al. Crystal structure of *E. coli* lipoprotein diacylglyceryl transferase. Nat Commun. 2016;7:10198. 26729647 10.1038/ncomms10198 PMC4728403

[20] Koch CA , Anderson D , Moran MF , et al. SH2 and SH3 domains: elements that control interactions of cytoplasmic signaling proteins. Science. 1991;252(5006):668‐674. 1708916 10.1126/science.1708916

[21] Ivanova ME , Fletcher GC , O'Reilly N , et al. Structures of the human Pals1 PDZ domain with and without ligand suggest gated access of Crb to the PDZ peptide‐binding groove. Acta Crystallogr D. 2015;71:555‐564. 25760605 10.1107/S139900471402776X PMC4356366

[22] Cheng HC , Skehan BM , Campellone KG , et al. Structural mechanism of WASP activation by the enterohaemorrhagic *E. coli* effector EspF(U). Nature. 2008;454(7207):1009‐1013. 18650809 10.1038/nature07160 PMC2719906

[23] Lu YJ , Zhang YM , Rock CO . Product diversity and regulation of type II fatty acid synthases. Biochem Cell Biol. 2004;82(1):145‐155. 15052334 10.1139/o03-076

[24] Lu X , Vora H , Khosla C . Overproduction of free fatty acids in *E. coli*: implications for biodiesel production. Metab Eng. 2008;10(6):333‐339. 18812230 10.1016/j.ymben.2008.08.006

[25] Lee H , DeLoache WC , Dueber JE . Spatial organization of enzymes for metabolic engineering. Metab Eng. 2012;14(3):242‐251. 21946160 10.1016/j.ymben.2011.09.003

[26] Abumrad N , Harmon C , Ibrahimi A . Membrane transport of long‐chain fatty acids: evidence for a facilitated process. J Lipid Res. 1998;39(12):2309‐2318. 9831619

