# Supplementary material for: Using the inner membrane of Escherichia coli as a scaffold to anchor enzymes for metabolic flux enhancement
Source: Eng Life Sci. 2023 Jan 10;23(2):e2200034. doi: 10.1002/elsc.202200034 (PMC9893748; doi:10.1002/elsc.202200034)
Supplement: Supplementary file 1 — Supporting Information [file ELSC-23-e2200034-s001.docx]

Supporting Information

Using the inner membrane of *Escherichia coli* as a scaffold to anchor enzymes for metabolic flux enhancement

You Wang, Yushu Wang, Yuqi Wu, Yang Suo, Huaqing Guo, Yineng Yu, Ruonan Yin, Rui Xi, Jiajie Wu, Nan Hua, Yuehan Zhang, Shaobo Zhang, Zhenming Jin, Lin He, and Gang Ma





Fig S1. Growth phenotypes of BL21/pETara-Anchor strain on LB agar media with the concentration of L-arabinose from 0 to 0.2% (w/v) and ampicillin concentration from 0 to 200 (μg/ml).


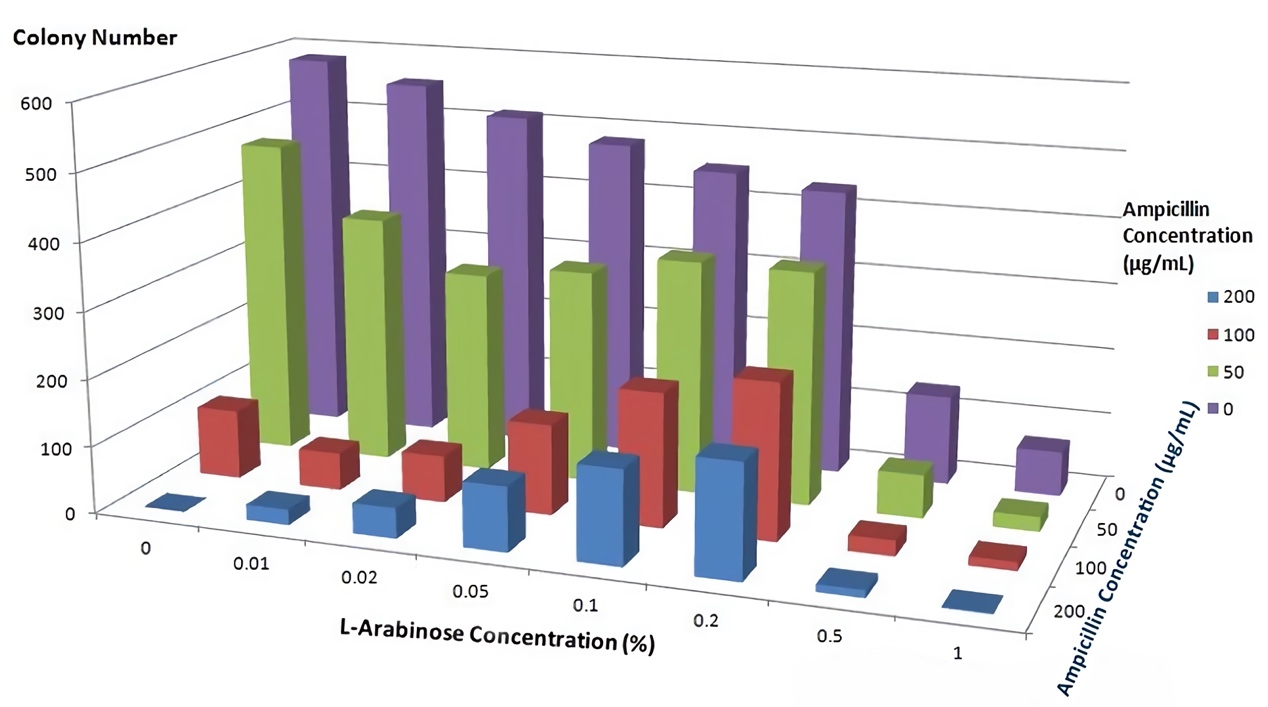


Figure S2. Growth condition of BL21/pETara-Anchor strain on LB agar media with different concentration of L-arabinose and ampicillin. Single colony is picked and cultivated at 37℃ until OD value reaches 0.7. Bacteria cultures are diluted by 1:100000 and coated onto plates containing graded concentration of 0-1% (w/v) L-arabinose and 0-200 μg/ml ampicillin. Growth condition is measured through counting colonies on plates.
